# Is it possible to reverse frailty in patients with chronic obstructive pulmonary disease?

**DOI:** 10.6061/clinics/2020/e1778

**Published:** 2020-10-21

**Authors:** Zhe Wang, Xiaojing Hu, Qingxiang Dai

**Affiliations:** Department of Geriatrics Medicine I, Qinghai University Affiliated Hospital, Xining, People’s Republic of China

**Keywords:** Chronic Obstructive Pulmonary Disease, COPD, Frailty, Pulmonary Rehabilitation

## Abstract

In recent years, frailty has attracted increasing attention from clinicians and health care workers. The influence of frailty on the elderly, especially those with chronic diseases of the respiratory system, is highly significant. Frailty is particularly more common in patients with chronic obstructive pulmonary disease (COPD). Frailty and COPD share many risk factors and pathophysiological mechanisms. As a comprehensive interventional method for chronic respiratory diseases, pulmonary rehabilitation is an important basic measure for the management of patients with COPD. Frailty in these patients can be reversed using pulmonary rehabilitation by targeting five components of the frailty phenotype at the entry point. The present review discusses the benefits of pulmonary rehabilitation in patients with COPD complicated by frailty and provides a theoretical basis for pulmonary rehabilitation treatment in this population. In addition, the timing of pulmonary rehabilitation is also addressed, with the prefrail stage being the “golden” period. The implementation of pulmonary rehabilitation must vary among individuals, and individualized treatment strategies will help maximize benefits.

## INTRODUCTION

With aging populations and changes in the environment and lifestyles, clinicians and health care workers have become increasingly interested in frailty (1). Frailty refers to a “risk state,” in which the body undergoes a disproportionate decrease in health subsequent to being exposed to an otherwise insignificant or relatively minor stimulus, which gives rise to negative health consequences and/or an augmented risk for death (2,3). As the study of frailty has deepened, researchers have discovered many health problems closely related to frailty (4). Fried et al. (4) proposed a frailty phenotype comprising five components: weight loss, exhaustion, weakness, slowness, and low physical function. Loss of physical function is prevalent among frail individuals, which in turn leads to impaired ability to perform the activities of daily living (5,6).

There is evidence that frailty may play an important role in the development of some chronic diseases and vice versa (7,8). In patients with chronic respiratory diseases (CRDs), frailty has demonstrated a close correlation with the common exacerbations of pulmonary disease, all-cause hospitalization, disability, and death (9). Chronic obstructive pulmonary disease (COPD) is a common disease that can be avoided and prevented, and is characterized by constant respiratory signs as well as airflow constraints (10). COPD patients constitute a common, vulnerable group of older individuals who may also exhibit frailty (5). COPD is a systemic disease in which common extrapulmonary manifestations include fatigue, weight loss, decreased physical activity, and muscle atrophy (11).

### Frailty and COPD

The relationship between COPD and frailty has not been fully elucidated; however, they share common risk factors (*e.g.*, age, smoking) and pathophysiological processes, which include systemic inflammation and endocrine dysfunction (12). Progressive increases in the severity of COPD and dyspnea will lead to a decline in physical activity, resulting in sarcopenia, dyskinesia, and, eventually, frailty.

The prevalence of frailty varies among COPD patients because of the different frailty assessment tools and components being evaluated (5,13,14). A meta-analysis by Marengoni et al. (15) revealed that the prevalence of frailty and pre-frailty in COPD patients was 20% and 56%, respectively. The authors found that individuals with COPD had twice the likelihood of becoming frail than did those without COPD (15). Additionally, frail COPD patients are predisposed to a larger risk for disability coupled with higher rates of health care use (5).

The impact of COPD with frailty on adults does not simply refer to an additive impact, as patients with COPD with frailty may be readmitted for an exacerbation of COPD within 90 days of discharge, thus invisibly contributing to healthcare costs (12). Research has suggested that the cumulative effect of frailty and respiratory impairment on mortality is greater in COPD patients than in individuals with normal lung activity (12). Individuals with respiratory impairment and frailty are at an approximately four-fold added risk for mortality (12). Galizia et al. (16) investigated mortality in elderly individuals with COPD and frailty. The frail COPD subjects had a higher mortality rate than did non-frail COPD subjects over a 12-year follow-up period (16).

### Frailty is reversible

A previous study reported that the likelihood of reversing frailty without intervention is minimal (8). Although there are no specific guidelines for treating frail COPD patients, prospective research by Maddocks et al. (11) appeared to indicate a way forward in that 73 frail COPD patients who completed an 8-week pulmonary rehabilitation (PR) program became non-frail (11). This suggests that frailty in patients with COPD can be ameliorated through PR, and, eventually, the prognosis of these patients can be improved. However, to the best of our knowledge, no prospective studies have fully elucidated the mechanism by which PR reverses the state of frailty in this patient population.

According to the definition by the American Thoracic Society and the European Respiratory Society, PR refers to “a comprehensive intervention based on a thorough assessment of the patient followed by individualized treatment, including but not limited to exercise training, education, and behavior change, aimed at improving the physical and mental status of patients with CRDs and at promoting long-term adherence to health-enhancing behaviors” (17). Since the introduction of PR programs, reassuring results have been achieved in the treatment of CRDs, and health care providers have gradually realized that PR constitutes a pivotal basic measure in managing individuals with COPD (18).

An entirely new model of PR, known as “interdisciplinary intervention,” is good news to frail COPD patients. Because of the similarities between COPD and frailty, we speculate that PR programs achieve the goal of reversing frailty by addressing the five components of the frailty phenotype, as described in the following sections.

### Weight loss

With the progression of illness, weight loss and muscle atrophy are usually observed in patients with COPD. Sarcopenia constitutes a pivotal component in the occurrence and development of frailty (19,20). COPD patients often exhibit decreased skeletal muscle mass and type I muscle fibers because of decreased appetite, decreased activity, and long-term hypoxemia. As such, the long-term effects of chronic inflammatory responses and hypercapnia further lead to skeletal muscle decomposition, anabolic imbalance, and oxidative damage of skeletal muscle, eventually leading to sarcopenia associated with the occurrence of frailty (21,22).

As part of a PR program, nutritional supplementation has been shown to be beneficial in improving body composition in patients with COPD, including elevation of body mass index (BMI), fat-free mass (FFM), and fat-free mass index (FFMI) (17). Moreover, there is also moderate-quality evidence indicating that nutritional supplementation is solely capable of promoting weight gain in malnourished COPD patients (23). Conversely, exercise training alone lowers body weight in those with advanced COPD, which is likely attributable to protein breakdown—particularly skeletal muscle protein—causing differing degrees of reduction not only in fat mass but also FFM (24,25). PR guidelines and a meta-analysis recommended combining health education, nutritional interventions, and exercise training for the purpose of improving body composition abnormalities in patients with COPD (17,23). According to a statement addressing nutritional management in COPD patients issued by the European Respiratory Association in 2014, it could be anticipated that if nutritional supplementation is combined with exercise training, malnourished COPD patients are likely to benefit maximally in terms of body composition (26). Several studies investigating nutritional supplementation in combination with exercise training interventions in COPD patients have shed light on the enhancements in BMI and FFM compared with baseline levels (27,28). Furthermore, adding nutritional supplements enhanced the compliance of participants engaged in PR (25). For these COPD patients, comprehensive PR is best included with—but not limited to—health education, nutritional intervention, and exercise training so that they do not experience the weight loss often associated with frailty.

### Exhaustion

Severe fatigue, which is insufficiently appreciated, manifests in more than one-half of COPD patients. This extrapulmonary sign is believed to be “the subjective perception of fatigue coupled with exhaustion and a lack of power occurring every day” (29-31). Fatigue is capable of significantly impacting not only the physical but also the mental well-being of patients with COPD, which is usually linked to anxiety, depression, and dyspnea (32,33). A four-year observational study investigating fatigue in patients with COPD confirmed this perspective (34). The report suggested that the detection rate of serious fatigue is doubled in these COPD patients despite the best care.

Investigations have revealed an improvement in fatigue among COPD patients following PR and that this improvement is likely attributed to PR addressing the risk factors that lead to fatigue, such as anxiety, depression, and dyspnea (33). In a systematic review, three randomized controlled trials (n=269) of comprehensive PR led to reductions in short-term anxiety and depression (35). Therewithal, a meta-analysis of 11 research studies reported that PR, being an effective intervention, contributed to significant improvement in anxiety and depressive symptoms in COPD patients (36). Although the above-mentioned studies lacked a description of the subjective fatigue of the participants, we speculate that subjective fatigue was improved after PR. Peters et al. (37) reported a significant improvement in checklist individual strength (CIS) subjective fatigue scores of patients with COPD in the control cohort subsequent to a 12-week PR program. Therefore, we speculate that the implementation of a PR program in prefrail or frail COPD patients with obvious symptoms of fatigue may alleviate—or even reverse—frailty by reducing the occurrence of symptoms such as anxiety, depression, and even dyspnea.

### Weakness

Individuals with COPD experience a loss of muscle strength, particularly in the lower limbs (38). Lower limb muscle dysfunction is recognized among the common extrapulmonary effects in those with COPD, and it causes a gradual decrease in the activities of daily living (39). Moreover, patients with COPD usually experience a loss of skeletal muscle strength owing to a vicious cycle of inactivity that also has a close correlation with the occurrence of frailty (5,40,41).

Resistance training is used as a mode of exercise in PR, in which local muscle groups are trained by repetitive lifting of relatively heavy loads, which can promote healthy aging in adults (42-45). Resistance training can be categorized into several different groups according to the number of joints involved: either multi-joint or single-joint exercises. In addition, multi-joint training demonstrated greater effectiveness in improving muscle strength when the total amount of work was consistent (46). When performing resistance training, sequential strength exercises are still recommended to optimize the quality of exercise intensity (large muscle group first, followed by small muscle group exercise; multi-joint, followed by single-joint exercise; and high-intensity, followed by low-intensity exercise) (45). Muscle atrophy represents a pivotal risk factor for falls in the elderly and particularly in frail COPD patients (47). Accordingly, the optimization of muscle strength constitutes a pivotal objective of PR in this population. Research has confirmed that resistance/strength training is capable of improving muscle strength in COPD patients, both at home and in a hospital or rehabilitation center (48,49). As a new means of PR, neuromuscular electrical stimulation may be a safe and productive method for improving muscle strength and exercise capacity in patients with moderate-to-severe COPD who are unable to engage in resistance training (50).

### Slowness

Gait speed is a suitable measure of physical functional capacity and is often associated with 6 min walk distance (6MWD). However, in patients with COPD, the 4 m gait speed (4MGS) test may be more practical in assessing frail individuals because more prolonged and distanced tests are difficult and impractical for those with advanced COPD (51-53). Gait speed has been shown to decline with increasing severity of COPD (51).

PR improves gait speed in many participants by increasing 6MWD. Although the duration of PR (walking as the primary exercise intervention, but also including health education, breathing training, and limb training) varied from 2 to 12 months, the mean 6MWD increased to varying degrees (62 m and 71.46 m, respectively) after completion of the program (54,55). This means that gait speed increased by at least 0.17 m/s, which is greater than the minimum clinical difference of 0.11 m/s in gait speed for 4MGS (56). There are also a small number of patients with COPD whose gait speed increases less than the minimum clinical difference value after PR; however, this does not prevent patients from benefiting from PR because it may improve survival outcomes (56,57). In short, these frail COPD patients can benefit from a comprehensive PR program that includes walking as an exercise.

### Low physical activity

In general, the majority of individuals worldwide do not meet the World Health Organization recommendations of a minimum of 150 min per week of moderate-to-vigorous or 75 min per week of vigorous physical activity, with sedentary behavior reaching global epidemic levels (58). Because of systemic manifestations of COPD, the number of individuals reaching these exercise targets may even be smaller. Among the COPD patient population, the average time spent sitting has been reported to be close to 7.5 h (445 min) per day, regardless of disease severity and level of lung function (59). Currently, there is evidence suggesting that sedentary behavior contributes to the risk for disability, together with a positive correlation with the development of frailty (60,61).

PR and its components, which aim to augment tolerance and enhance self-efficacy, could be regarded as a potentially reasonable intervention for the promotion of physical activity (17). Endurance exercise training in the form of cycling or walking is recognized as the most typically used exercise modality in PR (17). Nevertheless, it is likely difficult for frail COPD patients to achieve target intensities or training times. In this setting, low-intensity endurance training or interval training are suitable alternative options (62-65). Important health effects may occur with each increase in physical activity in the sedentary population, even when performing low-intensity physical activity (66). Similarly, replacing 30 min of sedentary behavior with an equal duration of low-intensity or moderate-to-vigorous intensity physical activity in a sedentary population lowered the prevalence of frailty by at least 16% (67). Briefly, therefore, “get moving and keep moving” is a good option to address frailty in patients with COPD.

## DISCUSSION

This review aimed to present the benefits of PR in frail COPD patients. We found that PR can reverse frailty by addressing five components of the frailty phenotype, namely, weight loss, weakness, exhaustion, slowness, and low physical function (Table 1). This provides a theoretical basis for PR to treat frail COPD patients.

Furthermore, the timing of initiation of PR has not been determined on the basis of the available literature. First, with the passage of time, patients in the prefrail stage tend to progress to the frail stage, and frailty is a “recession cycle” in which impairment in one parameter leads to a decline in other frailty parameters. Second, patients in the prefrail stage of frailty exhibited better tolerance to PR, a higher degree of completion than in patients in the frail stage, and a lower risk for falls when participating in PR programs. Finally, early identification of frailty is significant because timely and appropriate intervention(s) may prevent or delay functional decline, lower the risk for hospitalization and disability, and reduce mortality (68,69). Therefore, we believe that the prefrail stage is the “golden” (i.e., optimal) period for intervention.

Because of the heterogeneity of the COPD population, there is no uniform exercise prescription that targets a group of such patients. Currently, the commonly used methods are to formulate the walk training prescription on the basis of the results of the incremental shuttle walk test (ISWT)—an endurance training prescription based on a patient’s ability to complete 10 min of continuous training, the resistance training prescription based on patient tolerance, and the need for additional balance training based on whether there is a risk for falls. As a result, we believe that it is necessary to conduct a comprehensive evaluation of frail COPD patients before designing or prescribing a PR program. Strategies to tailor and modify PR programs to individual situations, therefore, are warranted and can increase the likelihood of optimal efficacy in individuals with the disease.

Most PR programs are currently conducted in hospitals or physical therapy centers. However, in developed countries (*e.g.*, Canada), participation in these programs is <1% (0.4%) despite the clear benefits of PR for those with CRDs (70). The lack of rehabilitation centers, high cost, and transportation are all issues that hinder participation in PR. There is low-to-moderate evidence that outpatient and home exercise training have the same effect on improving health-related quality of life (71). As such, home-based PR programs may be a promising solution.

## CONCLUSIONS

The present review illustrates the benefits of PR for COPD patients with frail/prefrail conditions, including improved body weight, increased FFM, increased muscle strength, improved gait speed, reduced subjective physical fatigue, and increased physical activity. We believe that PR can reverse frailty by addressing five components of the frailty phenotype and that the prefrail stage is the appropriate time to start such an intervention. A suitable PR program should be gradual and tailored to the characteristics of each patient. In the meantime, future research needs to devote close attention to the following two aspects: extend the observation time of COPD patients with frail/prefrail conditions who have completed PR as long as possible and perhaps find other benefits of PR for such patients and invest efforts into popularizing PR in COPD patients with frail/prefrail conditions.

## AUTHOR CONTRIBUTIONS

Wang Z and Hu X contributed equally to data curation, formal analysis, investigation, methodology, validation, and literature review of the study. Dai Q helped with data curation, formal analysis, methodology, and literature review of the study. Wang Z contributed to funding and wrote the original draft. All authors read and approved the final version of the manuscript to be submitted.

## Figures and Tables

**Table 1:** Summary of studies investigating the effects of pulmonary rehabilitation on components of the frailty phenotype.

First author, year (reference)	Study design	n	Pulmonary rehabilitation		Observation	Main result
			Frequency	Duration		
**Weight loss**						
van de Bool, 2017 (28)	RCT, double-blind	81	2-3/week	4 months	Total body mass, BMI, FM, SMM	After PR, body composition results demonstrated significantly increased body mass, skeletal muscle mass, and fat mass in the control group
Gurgun, 2013 (72)	RCT, prospective	46	2/week	8 weeks	Weight, BMI, FFMI	Combining oral NS with PR in depleted COPD patients improved body composition
**Exhaustion**						
Van Herck, 2019 (33)	Responder analyses	446	5 days/week	12 weeks	CIS subjective fatigue	After PR, the mean CIS-Fatigue score improved significantly and was clinically relevant
Gordon, 2019 (36)	Meta-analysis	734	2-5/week	4-16 weeks	HADS-A, HADS-D	PR conferred significant, clinically relevant benefits on anxiety and depression symptoms
Peters, 2017 (37)	Cluster analysis	160	5 days/week	12 weeks	CIS subjective fatigue	After PR, the mean CIS-Fatigue score significantly improved, and the improvement persisted one year later
**Weakness**						
Iepsen, 2015 (49)	Meta-analysis	331	2-3/week	3-12 weeks	Muscle strength	A combination of resistance and endurance training increased leg muscle strength in COPD
Vonbank, 2012 (73)	RCT	36	2/week	12 weeks	Muscle strength	Muscle strength improved in all groups
Chen, 2016 (50)	Meta-analysis	276	2-7/week	4-10 weeks	Quadriceps strength	NMES appeared to be effective in enhancing quadricep strength in patients with moderate-to-severe COPD
**Slowness**						
Li, 2019 (54)	Meta-analysis	414	2-7/week	2-12 months	6WMT	PR programs had beneficial influence in exercise function in elderly COPD patients
Kon, 2014 (56)	Longitudinal	301	2/week	8 weeks	4MGS	The 4MGS improved with PR
**Low physical activity**						
Probst, 2006 (63)	RCT	11	3/week	12 weeks	6WMT, W_max_	As expected, training W_max_ increased significantly after 12 weeks of exercise
Vogiatzis, 2002 (62)	RCT	36	40 min/day & 2 days/week	12 weeks	PWR, CRDQ	Interval training elicited substantial training effects.
Nagai, 2018 (67)	Cross-sectional	886	-	-	SB, LPA, MVPA, FP	Replacing 30 min of SB with an equivalent amount of LPA decreases the risk for frailty in older adults. Moreover, increasing LPA appeared more feasible than increasing MVPA in older adults, with substantial benefit

**Abbreviations:** BMI, body mass index; CIS, Checklist Individual Strength; COPD, chronic obstructive pulmonary disease; CRDQ: Chronic Respiratory Disease Questionnaire; FM, fat mass; FP, frailty phenotype; HADS-A, Hospital Anxiety and Depression Scale-Anxiety; HADS-D, Hospital Anxiety and Depression Scale-Depression; LPA, light-intensity physical activity; MVPA, moderate-to-vigorous intensity physical activity; NMES, neuromuscular electrical stimulation; NS, nutritional supplementation; PR, pulmonary rehabilitation; RCT, randomized clinical trial; SB: sedentary behavior; SMM, skeletal muscle mass; Wmax: baseline maximal workload; WR: peak work-rate; 4MGS, 4-meter gait speed; 6MWT, 6-min walk test.
